# The Oxford Shoulder Instability Score; validation in Dutch and first-time assessment of its smallest detectable change

**DOI:** 10.1186/s13018-015-0286-5

**Published:** 2015-09-17

**Authors:** Just A. van der Linde, Derk A. van Kampen, Loes W. A. H. van Beers, Derek F. P. van Deurzen, Caroline B. Terwee, W. Jaap Willems

**Affiliations:** Department of Orthopedic Surgery and Traumatology, Onze Lieve Vrouwe Gasthuis, Postbus 95500, 1090 HM Amsterdam, The Netherlands; Department of Orthopedic Surgery and Traumatology, Waterland Ziekenhuis, Purmerend, The Netherlands; Department of Epidemiology and Biostatistics and the EMGO Institute for Health and Care Research, VU Medical Center, Amsterdam, The Netherlands; Department of Orthopedic Surgery and Traumatology, De Lairesse Kliniek, Amsterdam, The Netherlands

**Keywords:** Validation, Oxford, Shoulder, Instability, Score, Dutch

## Abstract

**Background:**

The Oxford Shoulder Instability Score (OSIS) is a short, self-reported outcome measurement for patients with shoulder instability.

In this study, the OSIS was validated in Dutch by testing the internal consistency, reliability, measurement error, validity and the floor and ceiling effects, and its smallest detectable change (SDC) was calculated.

**Methods:**

A total of 138 patients were included. Internal consistency was calculated with Cronbach’s α. Reliability (test-retest) was calculated with the intraclass correlation coefficient (ICC). The measurement error was calculated (SEM), and the SDC was estimated in a subgroup of 99 patients that completed the re-test after a mean of 13 days (5–30 days). Construct validity was evaluated by comparing the OSIS with the Western Ontario Shoulder Instability index (WOSI), the Simple Shoulder Test (SST), the Oxford Shoulder Score (OSS), the Disability of the Arm, Shoulder, and Hand assessment (DASH), and the Short Form-36 (SF-36).

**Results:**

Internal consistency was good, with a Cronbach’s α of 0.88. The reliability was excellent, with an ICC of 0.87. The SEM was 3.3 and the SDC was 9 points (on a scale of 0–48). Regarding the construct validity, 80 % of the results were in accordance with the hypotheses, including a high correlation (0.82) with the WOSI. No floor or ceiling effects were found.

**Conclusions:**

The Dutch version of the OSIS showed good reliability and validity in a cohort of patients with shoulder instability.

**Electronic supplementary material:**

The online version of this article (doi:10.1186/s13018-015-0286-5) contains supplementary material, which is available to authorized users.

## Background

Shoulder instability is common in orthopedic practice; it generally affects young, active patients [1–3].

Research and evaluation of therapies for shoulder instability should focus both on objectively verifiable outcomes, such as the range of motion and re-dislocations, and on subjective functioning. A variety of patient-reported outcome measures (PROM) exist, some of which are specifically designed to reflect the patient’s subjective assessment of function. They enable the practitioner to detect functional changes in a standardized format. Because patients and doctors do not always agree on functional outcome after therapeutic interventions [4], PROMs have become increasingly important in assessing patient health status [5]. They can focus on general health; a physical domain or body part, such as the shoulder; or a specific condition or disease, such as instability [5–7].

The Oxford Shoulder Instability Score (OSIS) is a comprehensive questionnaire including 12 questions to assess shoulder instability. With a Cronbach’s α of 0.92, a Pearson correlation coefficient of 0.97 and measurement error of 5.7, the OSIS has proven to be valid and reliable, making it clinically important in patients with shoulder instability [8]. The OSIS was proven to be a useful outcome measure in several clinical studies [9–11], but it has not been translated and validated in languages other than English.

Translation and validation of internationally used PROMs will lead to culturally equivalent instruments and allow direct comparisons of national and international study results [12–14]. The aim of this study was to translate and validate the OSIS for the Dutch population and to evaluate its measurement properties according to the Consensus-based Standards for the selection of health Measurement Instruments (COSMIN) guidelines [15].

## Methods

### Translation procedure

After we obtained the official licence for the original English version, the OSIS was independently translated into Dutch by three native Dutch-speaking, medically educated translators. When they reached a consensus, a professional translator and a native English speaker (without a medical background) independently translated the version back into English; both were blinded to the first version and emphasized specifically on the linguistic aspects. Finally, the latter version was compared to the original text, composing a pre-final version. All items were agreed to be relevant for this patient population, and taken together, the items represented a comprehensive measurement of shoulder instability.

The pre-final version was checked for cross-cultural differences. It was subsequently completed by 13 patients with shoulder instability that were asked independently to assess the comprehensibility of all questions. These patients were not included in our final analysis.

### Patients and procedures

To assess the reliability and validity of the OSIS in the Dutch population, 154 patients with shoulder instability were recruited. Institutional approval was obtained by the local ethics committee; Institutional Review Board (IRB): METC, OLVG Hospital, Amsterdam, The Netherlands. Written informed consent was obtained from all participants.

We planned to include at least 100 patients, which is considered excellent for assessing measurement properties [15, 16].

A total of 154 patients with shoulder instability were included; all were diagnosed by one of the doctors in the outpatient clinic or the emergency department.

Patients were eligible to participate when they were 16 years or older and had been diagnosed with shoulder instability, based on their history and clinical examination. All patients were included on the ER or outpatient department of a hospital in Amsterdam. Exclusion criteria were an inability to master the Dutch language, a fracture in the glenoid, or a fracture in the humeral head. Hill-Sachs lesions and bony Bankart lesions were included. Tourists and temporary inhabitants of Amsterdam that were followed up in another clinic were also excluded, to avoid patient burden as a result of double follow-up.

All patients were assigned a study number and received either a web-based questionnaire, or alternatively, an identical paper questionnaire to complete at home. The order of administration was fixed. The web-based version required answers to all questions prior to submission. Missing values in paper submissions were completed in an interview by telephone.

Patients were asked to complete the questionnaire twice, without intervention. Both times, the questionnaire was either web-based or on paper. The repeated questionnaire was completed after a maximum interval of 5 to 30 days; this interval was considered long enough to forget prior answers, and short enough to assume an unchanged shoulder condition [17, 18].

### Oxford Shoulder Instability Score

The OSIS is a disease-specific PROM that was developed by Dawson et al. in 1999 in the UK for assessing the outcome of treatment for shoulder instability [8].

This 12-item questionnaire contained five response categories for each question. In the original scoring system, answers were scored from 1 to 5 points and summarized to a total score that ranged from 12 (least impaired) to 60 (most impaired). The scoring system was revised in 2009, in accordance with the revised scoring for the Oxford Shoulder Score (OSS), which originated in the same institute [19]. In the revised scoring system, answers were scored from 0 to 4, and the score was reversed; thus, the total score ranged from 0 (most impaired) to 48 (least impaired). We presented the results in terms of the new scoring system.

The OSIS was originally validated in 92 patients with shoulder instability that against the Rowe and Constant scores, with correlations of 0.51 and 0.56, respectively. The internal consistency (Cronbach’s α) was 0.92. The reliability was 0.97, calculated with a Pearson correlation coefficient. The measurement error was 5.7 points, calculated with the Bland and Altman method. No intraclass correlation coefficient (ICC) was calculated [8]. To date, no cross-cultural validation has been conducted.

### Validation instruments

The following instruments were solely used to assess the construct validity of the OSIS. No other data is used from these additional questionnaires. All instruments have been validated in Dutch, with good to excellent reliability and internal consistency [17, 20–23].

#### Western Ontario Shoulder Instability index (WOSI)

The WOSI is a disease-specific PROM for assessing the outcome of treatment for shoulder instability [24, 25]. Responses to the 21-item questionnaire were summarized in a total score, ranging from 0 or 0 % (no limitations) to 2100 or 100 % (extreme limitations).

It has been validated in Italian, German, Swedish, Japanese and Dutch [20, 26–30]. The Dutch version was validated using the same dataset as was used for the OSIS validation.

#### Simple Shoulder Test (SST)

The SST is a body-part-specific PROM [31]. It was designed to measure functional limitations of patients with general shoulder complaints. A cumulative score is calculated based on 12 questions (yes/no) and ranges from 0 (poor) to 12 (excellent shoulder function). It was validated against the American Shoulder and Elbow Surgeons (ASES) survey with a correlation of 0.81 [31].

#### Oxford Shoulder Score (OSS)

The OSS is a body-part-specific PROM. It was developed and validated for patients with general shoulder complaints [32]. Responses to the 12-item questionnaire were summarized to a total score that ranged from 12 (least impaired) to 60 (most impaired). This scoring system was revised in 2009 [19]. Currently, answers are scored from 0 to 4, and the summary is reversed; thus, the total score ranges from 0 (most impaired) to 48 (least impaired).

The OSS was originally validated against the Constant shoulder score and the SF-36 subscales [32]. Since that validation, it has been validated in Danish, Korean, Turkish, Italian, German, and Dutch [22, 33–37].

#### Disability of the Arm, Shoulder, and Hand (DASH) assessment

The DASH assessment is a body-part-specific PROM designed [38] to measure physical function and symptoms in patients with musculoskeletal disorders from any condition in any joint in the upper extremity.

Responses to the 30-item questionnaire are used to calculate the total score by averaging the item scores, subtracting 1, and multiplying the result by 25. The resulting score ranged from 0 (no disability) to 100 (extreme disability).

The DASH was shown to be reliable, valid, and responsive for patients with shoulder disabilities [39, 40].

#### Short form 36 Health Survey, version 1 (SF-36)

The SF-36 is a general health PROM that includes 36 questions for assessing the general health of patients with all kinds of disorders. It is the most widely used PROM for assessing general health [41]. It includes eight domains: physical function, social function, role limitations caused by physical problems (role physical), role limitations caused by emotional problems (role emotional), general mental health, vitality, bodily pain and perception of general health. Each domain has a total score of 0 (extremely poor) to 100 points (no complaint) [42].

The SF-36 was translated and validated in a Dutch general population, with a mean alpha coefficient across all scales and samples of 0.84. Previous studies have also validated the SF-36 specifically for shoulder complaints [43, 44].

### Assessments of measurement properties

#### Internal consistency and factor analysis

Internal consistency tells you to what extend different items within one questionnaire measure the same construct of interest (e.g. shoulder instability). Ideally, this score is high, indicating that all items measure the same construct. The internal consistency of the OSIS was assessed by calculating Cronbach’s α. For acceptable internal consistency, the Cronbach’s α should preferably be ≥0.7 [43].

Internal consistency can also be addressed using confirmatory factor analysis. See Additional file 1: Appendix 1.

#### Measurement error

Measurement error is the systematic, random error in the construct, which cannot be attributed to true changes in the patient’s condition [6]. When a score changes within the range of measurement error, it is not clear whether the change is a true effect of therapy or whether it should be attributed to measurement error.

Measurement error can be expressed as the standard deviation of repeated measurements in a single patient, referred to as the standard error of measurement (SEM). The SEM was calculated from the square root of the variance between the measurements and the error variance of the ICC. Subsequently, the SEM can be transformed into the smallest detectable change (SDC = 1.96*√2*SEM). The SDC represents the minimal change that a patient must show to ensure that the observed change is real, and not a measurement error [45]. The SDC is thus calculated; it is not derived from clinical observations following treatment.

#### Reliability

Since each instrument has a degree of uncertainty due to measurement error, reliability is defined as the degree to which the measurement is free from measurement error [6]. The reliability refers to the proportion of the total variance in the measurements that can be attributed to true differences between patients. Reliability was assessed by calculating the ICC, which was calculated with a two-way, mixed-effects model for absolute agreement. The mixed-effect model is used because a ‘fixed’ value (all questions remained unchanged during the whole cohort) is compared to a ‘random’ value (a cohort of patients was selected from all patients with shoulder instability). Scores ≥0.70 are considered adequate [45].

#### Construct validity

Construct validity reflects whether the instrument measures what it was designed to measure. In case of shoulder instability, do questions actually measure the typical complaints following shoulder instability (e.g. How much pain do you experience in your shoulder with overhead activities?)? In the absence of a gold standard for comparison, hypotheses are formulated that state the expected correlation between the investigated instrument and similar PROMs. In this study, the condition-specific OSIS was compared with the condition-specific WOSI (instability) and with the body-part-specific SST, OSS and DASH (shoulder). Finally, it was compared with several subscales of the original version of the SF-36 for measuring general health status. Pre-determined a priori hypotheses are stated in Table 1. These six hypotheses lead to a total of 42 correlations (or comparisons between correlations). The hypotheses were based on clinical experience, knowledge about several PROMs, and a consensus among the study investigators.

**Table 1:** Pre-determined hypotheses for testing the validity of the Dutch version of OSIS; expected correlations

	Expected correlations
1. OSIS and WOSI	≥0.7
2. OSIS and SST	≥0.6
3. OSIS and OSS	≥0.6
4. OSIS and DASH	≥0.6
5. Correlation between OSIS and body-part-specific PROMs (SST, OSS, and DASH) should be at least 0.1 higher than that between OSIS and the generic SF-36 subscales	
6. Correlation between OSIS and SF-36 physical function scale should be at least 0.1 higher than the correlations between OSIS and the other SF-36 subscales	

The highest correlation (≥0.7) was expected between the two disease-specific PROMs (OSIS and WOSI). High correlations (≥0.6) were expected between similar body-part-specific PROMs (OSIS and SST, OSS, and DASH). These correlation coefficients were expected to be at least 0.1 higher than the correlations between the OSIS and the more general subscales of the SF-36. Finally, because the OSIS predominantly measured physical function, we expected the correlation between the OSIS and the SF-36 physical function to be at least 0.1 higher than the correlations between the OSIS and the other SF-36 subscales.

Construct validity was considered good when at least 75 % of the results (correlations) were in accordance with our hypotheses [46].

#### Floor and ceiling effects

Floor and ceiling effects occur when more than 15 % of patients achieve the lowest or highest possible score, respectively [47]. Moreover, when a patient scores close to one of the extremes at baseline, a real change (defined as the SDC) could cross that extreme. Patients that score within the SDC-range from one of the extremes can thus be regarded as being at either their floor or ceiling too.

#### Statistical analyses

Statistical analyses were performed with SPSS software, version 18.0.0 (SPSS, Gorinchem, The Netherlands).

## Results

No major differences occurred between the OSIS translations into Dutch and back into English, no content- or linguistic-related difficulties were reported. The final version was considered free of cross-cultural inconsistencies; all questions are applicable to the Dutch population.

**Fig. 1:**
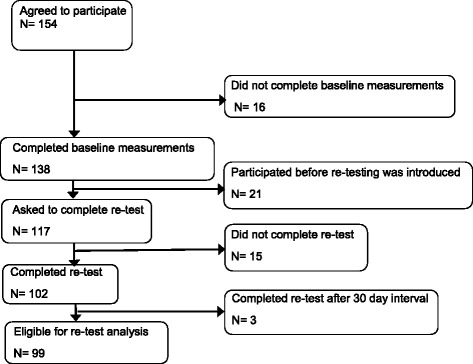
Flowchart of selection of patients that participated in the study

Figure 1 presents the selection of participating patients. One hundred and thirty-eight patients with shoulder instabilities completed the first questionnaire; 99 patients were eligible for the second questionnaire. The shoulder function, presented as the mean and SD scores of the WOSI, SST, OSS and DASH, did not differ significantly between the two measurements.

The demographic data and mean PROM scores are summarized in Table 2.

**Table 2:** Demographic data of patients completing baseline and the reliability cohort

		Baseline assessment	Reliability cohort
		*N* (%)	*N* (%)
Mean age, year (SD)		32 (12)	32 (14)
Gender (male)		98 (71 %)	66 (66 %)
Dislocated shoulder			
Right		72 (53 %)	54 (55 %)
Left		59 (43 %)	40 (40 %)
Both		6 (4 %)	5 (5 %)
Dominant side dislocated		73 (53 %)	53 (54 %)
Time first dislocation to completion OSIS			
<1 month		8 (6 %)	8 (8 %)
1–6 months		21 (15 %)	17 (17 %)
>6 months–2 years		40 (29 %)	25 (25 %)
> 2 years		67 (49 %)	49 (50 %)
Sports-related traumatic instability		71 (54 %)	47 (47 %)
WOSI	(100–0)^a^	46.0 (22.3)	45.7 (24.2)^b^
SST	(0–12)^a^	8.8 (3.1)	8.8 (3.2)^b^
OSS	(48–0)^a^	23.7 (7.8)	22.8 (8.3)^b^
DASH	(100–0)^a^	22.2 (16.7)	22.7 (18.3)^b^

^a^Ranges reflect most impaired to least impaired function

^b^No significant change in shoulder function (WOSI, SST, OSS, DASH) was observed at re-test compared to the baseline assessment

### Internal consistency and factor analysis

For all 138 patients that completed the OSIS at baseline, the Cronbach’s α was 0.88, indicating good internal consistency.

### Reliability

The mean time between the completion of the first and second questionnaires was 13 days (5–30). Table 3 presents the scores of the tests and re-tests and the ICC with a 95 % confidence interval (ICC is 0.87 (0.82–0.91). These results indicate excellent reliability.

**Table 3:** Test-retest reliability (ICC), standard error of measurement (SEM) and smallest detectable change (SDC) for the OSIS

	Baseline	Re-test	Change	SEM	SDC	ICC (95 % CI)
	Mean (SD)	Mean (SD)	Mean (SD)			
New scoring system	27.2 (9.3)	27.6 (9.7)	−0.4 (4.8)	3.3	9.0	0.87 (0.82–0.91)

Scores are expressed according to the new scoring system. This analysis included 99 patients that completed the baseline and retest evaluations

### Measurement error

The SEM was 3.3, which resulted in a SDC of 9.0 points, indicating that a patient has to show a change of 9.0 points to ensure the detection of a true change. This is 19 % of the total range.

### Construct validity

The observed correlation results are summarized in Table 4. In total, 80 % of the results were in accordance with our hypotheses. The hypotheses were confirmed for the correlation between the OSIS and the other instability-specific WOSI (0.82; ≥0.7 expected) and the correlations between the OSIS and the shoulder-specific SST, OSS and DASH (0.69, 0.76, and 0.79, respectively; ≥0.6 expected). The hypothesis was partly confirmed for the strength of the correlation between the OSIS and the SF-36 subscales.

**Table 4:** Observed correlations for testing the validity of the Dutch version of OSIS

	Observed correlations
1. OSIS and WOSI	0.82
2. OSIS and SST	0.69
3. OSIS and OSS	0.76
4. OSIS and DASH	0.79
5. OSIS and SF-36 subscales:	
Physical function	0.65
General health	0.31
Social function	0.56
Vitality	0.39
Mental health	0.20
Role emotional	0.41
Role functional	0.69
Bodily Pain	0.78

### Floor and ceiling effects

No patients scored minimum or maximum scores. At most, 12 % of patients scored within the SDC-range for the lowest possible score. The results are presented in Table 5.

**Table 5:** Floor and ceiling effects of the OSIS scoring system

Scoring system	Absolute floor	Absolute ceiling	SDC	SDC-range from		% of patients scoring within SDC range	
				Floor	Ceiling	Floor	Ceiling
New (48–0)^a^	No	No	9.0	48–39	9–0	12 %	4 %

From left to right, the new scoring system with the ranges and the absolute floor and ceiling scores are presented. The smallest detectable change (SDC) and the percentage of scores that fell within the SDC-range for both extremes

^a^Ranges reflect least impaired to most impaired function

## Discussion

There is a growing interest in PROMs for both clinical and research purposes to supplement clinical outcome measures. To our knowledge, this is the first study to validate the OSIS in a foreign language and the first to report the measurement error and evaluate floor and ceiling effects.

The results show a high internal consistency (Cronbach’s α = 0.88); it was only slightly lower than that described in the original article (Cronbach’s α = 0.91 at pre treatment [*n* = 92] and 0.92 at follow-up [*n* = 64]). Compared to other Dutch-validated PROMs, our Cronbach’s α for the OSIS was higher than that of the SST (0.78) and lower than that of the OSS (0.92) [17, 22].

Considering the content of the questions, it is clear that the OSIS measures several constructs, such as pain, physical-, social-, and role functioning, frequency of dislocation and worries.

The reliability was addressed with a test-retest sample in 99 patients with a mean interval of 13 days (5–30) and showed an ICC of 0.88. This was lower than the 0.97 that Dawson et al. described after a 24-h interval in 34 patients; nevertheless, 0.88 is considered a very good ICC.

To our knowledge, the measurement error (SDC) of the OSIS has not been reported previously. Our SDC value showed that, to determine a treatment effect, one must find a difference of at least 9 points between two scores from an individual patient to ensure that the difference was not due to measurement error [48].

To assess the construct validity, Dawson et al. calculated correlations with the Rowe and Constant scores. However, the Rowe and Constant scores are not PROMs but observer-based measurement instruments. Moreover, the Constant score is not considered applicable to shoulder instability [49, 50]. Therefore, the construct validity was assessed by calculating correlations with the WOSI, the SST, the OSS, the DASH and the SF-36 subscales. With 80 % of the results in accordance with our hypotheses, the construct validity was considered good. The highest correlation (0.82) was observed between the two instability-specific PROMs (OSIS and the WOSI).

A high correlation was observed with the DASH (0.79), which addresses daily activities more specifically than the OSIS. However, many questions overlapped such as ‘putting on a pullover sweater’ (DASH) and ‘during the last three months, have you had any trouble (or worry) dressing, because of your shoulder?’ (OSIS). This similarity might explain the high correlation between the two instruments.

The OSIS was more closely correlated with the SF-36 subscales ‘pain’ (0.78) and ‘role physical’ (0.69) than with the subscale ‘physical function’ (0.65). These correlations were comparable to those described by Dawson et al. This may indicate that, in addition to physical function, the OSIS measures aspects of pain and role limitations due to physical problems.

In previous studies, floor and ceiling effects were not addressed. In this study, no patient had the maximum or minimum score. The estimation of the smallest detectable change indicated that the baseline patient scores should ideally be at least 9 points different from the extremes. That margin would enable detection of improvements and deteriorations that are distinct from measurement errors at follow-up. At most, 12 % of patients scored within the SDC-margin; thus, these scores were less than the commonly used cut off of 15 % [47].

A strong aspect of this study was the large size of our patient population without missing values.

Conversely, an unavoidable limitation of this study was the total number of questions posed to the patients. Completing six questionnaires at once requires considerable time and concentration, and patients might have digressed or lost focus. Also, although web-based versions have many advantages over paper versions such as an increased follow-up ratio and prevention of missing data, validation of digital formats should still be performed. Here, the results are expressed according to the new scoring system. It is important to be aware of the changed scoring system, and we recommend that future studies should specify the scoring system used.

Finally, for future studies, it would be very interesting to determine responsiveness and the minimal important change (MIC) of the OSIS. This information can be used to determine whether the observed change is important to the patient and to calculate the percentage of patients that report changes greater than the MIC (responders) in each arm of a trial. Then, the percentage of responders can be compared between groups [51].

## Conclusion

This study found that the Dutch version of the OSIS was a reliable outcome measure in patients with shoulder instability, with a Cronbach’s α of 0.87 and an ICC of 0.87. In addition, the construct validity was considered good. Comprising 12 questions, the OSIS is user-friendly and can be easily administered. Furthermore, in the absence of floor or ceiling effects, it is a valuable PROM in clinical practice. Patients need to change at least 9 points to ensure that the difference is not due to measurement error.

The Dutch version of the OSIS can be acquired by its managing institution, Isis Outcomes, Isis Innovation Ltd, holding its copyright (http://isis-innovation.com/outcome-measures/oxford-shoulder-instability-score-osis/).

## Additional file

Additional file 1: Appendix 1. Confirmatory factor analysis to assess internal consistency. (DOCX 19 kb)
