# Supplementary material for: The Oxford Shoulder Instability Score; validation in Dutch and first-time assessment of its smallest detectable change
Source: J Orthop Surg Res. 2015 Sep 17;10:146. doi: 10.1186/s13018-015-0286-5 (PMC4574347; doi:10.1186/s13018-015-0286-5)
Supplement: Additional file 1: Appendix 1. — Confirmatory factor analysis to assess internal consistency. (DOCX 19 kb) [file 13018_2015_286_MOESM1_ESM.docx]

**Appendix 1**

Methods

Factor loadings represent the correlation between the items in the questionnaire and the factors (the underlying dimensions, such as work or lifestyle). Factor loadings are generally considered to be meaningful when they exceed 0.30 or 0.40 [1]. We considered factor loadings of at least 0.50 appropriate. The Comparative Fit Index (CFI), Tucker-Lewis Index (TLI), and the Root Mean Square Error of Approximation (RM­SEA) were used as measures for model fit. A CFI and TLI of > 0.95 and a RMSEA of < 0.05 were considered as adequate fit. For a moderate fit, values > 0.90 and < 0.08 were used. Because the model didn’t fit well (see results section), an additional exploratory factor analysis was performed in SPSS, using Varimax rotation.

Analogous to Pearson's r, the squared factor loading is the percent of variance in that indicator variable explained by the factor.

Confirmatory factor analysis for categorical items was performed in Mplus using the method of weighted least squares with mean and variance adjustment (WLSMV). We examined factor loadings and model fit.

Results

Factor loadings are presented in Table 6. A one-factor model fitted the data not good (CFI was 0.901, TLI was 0.879 and RMSEA was 0.147), suggesting multiple contributing factors. Item 1 had a low factor loading of 0.255 and was removed to evaluate the effect on the model. No other items were removed. Removing item 1 did not improve the model (CFI 0.898, TLI 0.873, RMSEA 0.162). An exploratory factor analysis resulted in a three-factor model, explaining 63.6% of the variance, with items 2,3,4 and 7 loading on the first factor, items 8 through 12 loading on the second factor and items 1,5 and 6 loading on the third factor. This model was not clearly interpretable. A 2-factor model was also considered (explaining 55,0% of the variation), but this did not lead to a clearly interpretable model, with multiple items loading on both factors.

Table 6. Factor loadings of the OSIS. Estimate and standard error (SE).

| Question | Estimate | SE |
| --- | --- | --- |
| 1 | 0.255 | 0.071 |
| 2 | 0.736 | 0.039 |
| 3 | 0.592 | 0.051 |
| 4 | 0.822 | 0.029 |
| 5 | 0.756 | 0.037 |
| 6 | 0.823 | 0.029 |
| 7 | 0.714 | 0.047 |
| 8 | 0.736 | 0.041 |
| 9 | 0.605 | 0.067 |
| 10 | 0.760 | 0.038 |
| 11 | 0.727 | 0.038 |
| 12 | 0.612 | 0.059 |

Discussion

This is the first study that evaluated the dimensionality of the OSIS, dimensionality or factor loadings were not addressed in the original paper. Because the one-factor model did not fit the data good, the scale is not uni-dimensional. Summary scores should therefore be interpreted with caution. Especially when interpreting change scores because changes can be due to different changes in any of these constructs. A change of 10 points in one patient may therefore not be comparable to a change of 10 points in another patient.

Reference

[1] Hu LT, Bentler PM. Cut-off criteria for fit indexes in covariance structure analysis: Conventional criteria versus new alternatives. 1999. p. 1-55.
